# Promoting Mentalizing in Pupils by Acting on Teachers: Preliminary Italian Evidence of the “Thought in Mind” Project

**DOI:** 10.3389/fpsyg.2016.01213

**Published:** 2016-08-31

**Authors:** Annalisa Valle, Davide Massaro, Ilaria Castelli, Francesca Sangiuliano Intra, Elisabetta Lombardi, Edoardo Bracaglia, Antonella Marchetti

**Affiliations:** ^1^Research Unit on Theory of Mind, Università Cattolica del Sacro CuoreMilan, Italy; ^2^Department of Psychology, Università Cattolica del Sacro CuoreMilan, Italy; ^3^Department of Humanities and Social Sciences, Università degli Studi di BergamoBergamo, Italy

**Keywords:** mentalizing, theory of mind, training, teacher-pupil relationship, TiM Project, resilience

## Abstract

Mentalization research focuses on different aspects of this topic, highlighting individual differences in mentalizing and proposing programs of intervention for children and adults to increase this ability. The “Thought in Mind Project” (TiM Project) provides training targeted to adults—teachers or parents—to increase their mentalization and, consequently, to obtain mentalization improvement in children. The present research aimed to explore for the first time ever the potential of training for teachers based on the TiM Project, regarding the enhancement of mentalizing of an adult who would have interacted as a teacher with children. For this reason, two teachers – similar for meta-cognitive and meta-emotional skills - and their classes (*N* = 46) were randomly assigned to the training or control condition. In the first case, the teacher participated in training on the implementation of promotion of mentalizing in everyday school teaching strategies; in the second case the teacher participated in a control activity, similar to training for scheduling and methods, but without promoting the implementation of mentalization (in both conditions two meetings lasting about 3 h at the beginning of the school year and two supervisions during the school year were conducted). The children were tested by tasks assessing several aspects of mentalization (second and third-order false belief understanding, Strange Stories, Reading the mind in the Eyes, Mentalizing Task) both before and after the teacher participate in the TiM or control training (i.e., at the beginning and at the end of the school year). The results showed that, although some measured components of mentalization progressed over time, only the TiM Project training group significantly improved in third order false belief understanding and changed - in a greater way compared to the control group – in two of the three components of the Mentalizing Task. These evidences are promising about the idea that the creation of a mentalizing community promotes the mentalization abilities of its members.

## Introduction

Mentalizing and theory of mind are two constructs often used interchangeably, although they cannot be considered perfectly overlapping (Sharp and Venta, 2012). Analyzing the studies in this area, it emerges that mentalizing is the construct more often used in the “clinical framework,” whereas theory of mind is the construct more often used in the “cognitive and socio-constructivist one.” This study is in line with those theoretical positions that highlight the similarities rather than the differences between these two concepts. We also explicitly refer to the literature that stresses the relational co-construction of children’s theory of mind thanks to their relationships with significant caregivers (Dunn et al., 1991; Dunn, 1994). The importance of this interpersonal dimension is largely responsible for the individual differences in the developmental paths of mentalization. Mentalization, or mentalizing (Allen, 2006), is a mental activity consisting in the ability to understand and to interpret human behavior on the basis of intentional mental states as beliefs, desires, intentions, goals, and emotions (Bateman and Fonagy, 2006; Fonagy, 2006; Choi-Kain and Gunderson, 2008; Fonagy and Allison, 2012). Mentalizing is an imaginative activity including a wide range of cognitive operations about one’s own and others’ mind, such as interpreting, inferring, remembering and so on (Allen, 2003). Choi-Kain and Gunderson (2008) identified three dimensions of the construct of mentalization: (1) the functioning (implicit and explicit), (2) the objects (self and others), and (3) the aspects (cognitive and affective). The first dimension refers to the fact that mentalization can be an implicit, automatic, and pre-reflective process when the subject acts on the basis of an intuition about mental contents (for example, during a conversation), but also an explicit, symbolic, and conscious activity when the individual intentionally reflects about the mind (for example, in psychotherapy; Allen et al., 2008). The second dimension indicates that mentalizing happens during interactions (Allen, 2006) where people reflect about the minds of all the participants of the social exchange. The third dimension highlights the fact that reasoning about intentional mental states is usually cognitively focused and affectively laden; the cognitive and affective aspects are closely connected. Moreover, the mentalizing process integrates the ability to reason about the epistemic mental contents and about emotions. Finally, the developmental model suggested by Allen et al. (2008) argues that the mentalization process is rooted in the attachment relationship established with the first caregiver in infancy and early childhood.

The concept of mentalization that Fonagy (1991) proposed derives both from the psychoanalytic term “reflective functioning,” and from the psychological construct of “theory of mind” (Choi-Kain and Gunderson, 2008). Based on psycho analytic work with borderline patients, a Mentalization Based Treatment (MBT) was created (Allen and Fonagy, 2006; Bateman and Fonagy, 2006, 2013): it is a clinical treatment designed to improve mentalization processes, which is impaired in these individuals. Recently, MBT has been adapted and applied to other clinical or atypical situations, including substances abuse, eating disorders, antisocial personality disorder, parental relationships at risk (Bateman and Fonagy, 2011), families with adopted children (Muller et al., 2012), and self-harm in adolescence (Rossouw and Fonagy, 2012). On the basis of the positive effects obtained from MBT in increasing mentalizing abilities in the above-mentioned situations, in recent years several researchers have been developing programs of intervention for non-clinical settings, such as schools. For example, Twemlow and colleagues (2005a,b) applied the mentalization principles in the Peaceful Schools Program, with the aim to create mentalizing school communities to reduce violence and bullying. The authors illustrated the two key components of their approach: (1) violent individuals and communities are impaired in mentalization, and (2) power dynamics involving these individuals and their communities tend to further reduce mentalization abilities. “*The difference between a violent and a non-violent community must be the degree to which the implicit social conventions are structured to encourage all participants to be aware of the mental states of others*” (Twemlow and Sacco, 2012, pp. 195–196). The main components of the Peaceful Schools Program are the following: (1) positive climate campaigns, stimulating and supporting the awareness of mental states and their role in violent contexts; (2) classroom management; i.e., training teachers to not use coercive discipline, but rather to refer to their mentalization abilities and to those of children; (3) peer and adult mentorship; i.e., training other adults to become mentors, able to intervene in a mentalistic way during violent episodes outside the classroom; (4) the “gentle warrior physical education program”; i.e., teaching children physical self-control in violent situations (a low activation of the body allows high activation of the mind); and (5) reflection time; i.e., the introduction in the classrooms of a 10 min period at the end of each day devoted to talking, from a mentalistic point of view, about the trend of the day and any situations of violence that occurred. The evaluation of the Peaceful Schools Program, longitudinally applied to children aged 8–11 years are encouraging (Fonagy et al., 2009). In contrast with traditional school psychiatry consultation and with usual treatment at school, this program moderated the increase of aggressiveness typical of this age period, the victimization phenomena, and the decline in empathy. Additionally, the program decreased the number of self-reported aggressive acts and aggressive bystanding.

Another proposal of the educational application of mentalization is the “Thought in Mind Project” (TiM Project), also named “Resilience Program,” created by Bak (2012). The TiM Project shares with the Peaceful Schools Program the assumption that the creation of a mentalizing community promotes the mentalization abilities of its members. Furthermore, it claims that in these type of communities mentalizing children can develop several strategies to react to the difficulties in their life, thus increasing their resilience (see Stein, 2006). This approach is also in line with the recent rethinking of resilience within a developmental systems framework, that claims – among other things – “the possibility of changes that spread across domains and levels through the many interactions of systems” (Masten, 2016, p. 301). The TiM Project addresses mentalization, resilience, and self-control concepts using simple language, metaphors, pictures, and short movies available on a dedicated website^1^. Clinicians or researchers propose and explain these materials to a target group (usually teachers and/or parents), who then use the materials as they deem most appropriate for their condition. A follow-up supervision is sometimes provided. An exploratory pilot study (Bak et al., 2015) proposed the TiM Project to the staff members of a social club for adolescents with disruptive behavior in a low income urban area in Denmark. Results showed that as a consequence of the TiM Project training, the yearly frequency of situations where the staff members of the club had to use physical force to solve high-risk conflicts among adolescents decreased significantly. The mental health of the staff increased and the methods introduced by this project continued to be used by the majority of the staff 3 years later.

The TiM Project training aims to clarify those cognitive processes strongly impregnated with mental contents through a metacognitive approach related to both emotional and epistemic contents. In addition, the training emphasizes the relational dimension, because it proposes an intervention directed to the caregivers that is likely to have a positive and long-term effect on children or adolescents. It may be interesting also to consider some indicators of the potential changes in children’s mentalizing ability. In our opinion, the psychological construct that fits this goal is the theory of mind. The reason is threefold: (1) it is a key component of mentalizing, (2) it has been explored through a broad and substantial range of tasks, and (3) its development can be supported by training specifically designed for this purpose.

Theory of mind is the ability to understand mental states (intentions, desires, thoughts, and beliefs), and to predict one’s own and others’ behavior on the basis of these understandings (Premack and Woodruff, 1978). Theory of mind develops during childhood and continues to evolve in adolescence (Valle et al., 2015) and adulthood (Apperly et al., 2009; Sommerville et al., 2013). According to a socio-constructivist approach, theory of mind emerges within contexts of social interactions, thanks to the participation in social exchanges (Astington and Olson, 1995; Carpendale and Lewis, 2004). In this theoretical perspective, an interesting construct that focuses on the relational potential in the mother–child dyad in supporting the development of theory of mind is mind-mindedness. It is the maternal proclivity to consider infants as intentional agents with mental states and to interact with them on the basis of such a belief (Meins et al., 2002, 2003). In this regard, it was highlighted that maternal mind-mindedness, operationalized as the ability to individuate and comment appropriately on their 8-month-olds’ internal states, was negatively related to children’s externalizing and internalizing behaviors specifically in low socioeconomic status families (Meins et al., 2013). Furthermore, mind-mindedness appears to be an important aspect of personal relationships rather than a trait-like quality (Meins et al., 2014). In this sense, it is likely that adults—supported in the development of activities of mentalization—may find it easier to engage in mentalization-oriented relationships. This evidence provides support to the implementation of the TiM Project, whose strong point is the involvement of the adults who take care of the children in the educational setting. Moreover, many research studies have shown that high levels of theory of mind are linked to different abilities, such as social competences (Jenkins and Astington, 2000; Razza, 2009), prosocial behaviors (Caputi et al., 2012), academic results (Malecki and Elliot, 2002; Lecce et al., 2011), and attribution of intentions in different daily situations (for example, attribution of fair or unfair intention during economical exchanges; Castelli et al., 2010, 2014). In light of these findings, several interventions have been constructed and evaluated in order to implement theory of mind in children. In typical development, different types of training positively affect theory of mind abilities in the short and medium term (Slaughter and Gopnik, 1996; Kloo and Perner, 2008; Grazzani and Ornaghi, 2011; Lecce et al., 2014; Ornaghi et al., 2014; Grazzani et al., 2016). In the case of learning disorders (Ashcroft et al., 1999) and intellectual disabilities (Adibsereshki et al., 2014), theory of mind training improved reflective and social skills. To construct and directly evaluate such training and its effects on the psychological development of children, classical and advanced theory of mind tasks are used. The possibility to rigorously evaluate the effect of these different types of training using psychological tasks has supported our idea that it is possible to realize a similar assessment in the TiM Project, which has been subjected only to indirect evaluations thus far (Bak et al., 2015). Any confirmation of the validity of the TiM project would be particularly interesting. In fact, this training is aimed at teachers and supports them in the implementation of strategies for the development of children’s mentalizing. The effects of this training are therefore indirect, as the aim is to support the children through an intervention involving teachers. If effective, the potential is considerable: maximum efficiency with low costs (since the teachers can use these strategies with all the children with whom they come into contact), and a greater likelihood of generalization and persistence of acquired skills due to the high integration of support practices to mentalizing within normal teaching strategies.

### Aims and Hypotheses

This research aimed to evaluate for the first time the efficacy of the TiM Project on a group of 10-year-old pupils. The hypothesis was that children whose teacher participated in the TiM Project training would improve theory of mind and mentalization styles more than a control group of children whose teacher participated in a training without mentalistic contents.

## Materials and Methods

### Participants

Forty-six ten-year-old children belonging to two school classes and the respective two teachers who spend more time with the class during the school year took part in the study. The two school classes were randomly assigned to the study groups: the TiM Project training group (*N* = 23, *M* = 10.26 years, *SD* = 3.16 months; 10 boys, 13 girls) and the control training group (*N* = 23, *M* = 10.23 years, *SD* = 5.16 months; 13 boys, 10 girls). All children were Italian and of middle socioeconomic status based on the parents’ education and socioeconomic levels. Children were not clinically referred for any cognitive or learning difficulties and were neither referred to social services nor reported by teachers for learning and socio-relational difficulties. The children were tested for those skills on which we hypothesized that the TiM Project training with the teachers would have a positive effect. The two teachers who participated in the study were both female, 34 and 35 years of age, and had a master degree and 10 years of working experience at the school. The teachers, depending on the class, participated in either the meetings for TiM project training or the meetings for the control group training.

### Tasks and Training

All children were evaluated by the following tasks in both the pre- and post-training phases (i.e., at the beginning and at the end of the school year, which was approximately a 6-month interval between the two phases).

#### Mentalizing Task

The Mentalizing Task (Sharp et al., 2007; Di Terlizzi, 2010) evaluates children’s mentalizing attributional styles in everyday life situations. The styles include the following: overly negative (ON), a cognitive mentalizing bias characterized by a global, negative, and stable self-attribution of the causes of social situations (“They would think nobody likes me”) typical of children with symptoms of depression and anxiety (Quiggle et al., 1992; Barrett et al., 1996); overly positive (OP), a cognitive mentalizing bias characterized by a global, positive, and stable self-attribution of the causes of social situations (“They would think I’m cool not to play silly games with the rest of the kids”) typical of aggressive children (David and Kistner, 2000) idealizing their own competence in interpersonal relationships; rational or neutral (R), a non-self-referent, non-stable type of interpretation of social situations (“They would think I’m just sitting down to have a think and a rest”) typical of children with a helpful, functional, and adaptive coping style. This forced-choice task, which lasts 10 to 15 min per child, included 15 stories and vignettes about social situations that happen at school to a certain child. At the end of each story, the researcher asked the participant the following: “Imagine you are [*the character*]. If you were, what do you think the other kids would be thinking about you?” The participant can choose among three options that reflect one of three mutually exclusive categories: ON, OP, or R that represent the three final variables. Each variable score can range from 0 to 15.

#### False Belief Tasks

To test children’s cognitive theory of mind competence we used two second-order false belief tasks (second FBTs; Sullivan et al., 1994; Astington et al., 2002; Liverta Sempio et al., 2005) and a third-order false belief task (third FBT; Valle et al., 2015), all based on the unexpected transfer paradigm. The two second FBTs were the Look Prediction version (LP) and the Say Prediction version (SP) (Sullivan et al., 1994; Astington et al., 2002; Liverta Sempio et al., 2005). In the LP and SP versions there are two control questions, two false belief questions, and a justification question. In the third FBT there are two control questions, a second-order false belief question with its justification, and a third-order false belief question with its justification. We attributed 1 point for each correct answer and 0 points for each wrong answer. The total score range is 0–2 for the second FBT and 0–2 for the third FBT. Two raters independently coded 33% of the responses at pre- and post-test and inter rater agreement was established using Cohen’s Kappa. This agreement was very high for both the second FBTs (at pre-test, LP: κ = 0.92; SP: κ = 0.89; at post-test, LP: κ = 1; SP: κ = 0.90) and the third FBT (at pre-test, κ = 0.92; at post-test, κ = 0.93).

#### Strange Stories

The Strange Stories (Happé, 1994) evaluate the application of theory of mind ability in everyday social situations. This task consists of 24 short stories where the protagonist does or says something strange, in order for the participant to explain the character’s strange behavior or provide a statement referring to the mental contents of the protagonist. As a control task the Physical Stories were used, in which in order to explain the character’s behavior or provide a statement, the participant has no need to refer to the mental contents of the character. In the present research we selected four Strange Stories (concerning sarcasm, double bluff, persuasion, and contrary emotions) and four Physical Stories. Two Strange Stories have one question, whereas the other two Strange Stories have two comprehension questions. Furthermore, each story has a justification question. Each comprehension question is scored 1 if correct and 0 if wrong. The justification question is scored 2 if correct and has an explicit answer, 1 if partially correct, and 0 if wrong. The total score range is 0–18. Each Physical Story has a comprehension question coded 2 if correct and has an explicit answer, 1 if partially correct, and 0 if wrong. The total score range is 0–8. Two raters independently coded 33% of the responses at pre- and post-test and inter rater agreement was established using Cohen’s Kappa. This agreement was very high for both the Strange Stories (at pre-test, κ = 0.89; at post-test, κ = 0.90) and the Physical Stories (at pre-test, κ = 0.92; at post-test, κ = 0.98).

#### Reading the Mind in the Eyes Test-Child Version

To test the affective component of theory of mind we used the Reading the Mind in the Eyes Test-Child Version (RMET; Baron-Cohen et al., 2001; Castelli, 2010) that requires the attribution of mental states to other people by observing the eye region of their face. The test comprises 28 pictures of the eye region of different people. The participant has to choose among four options the one that best represents what the character is thinking or feeling. Only one option is correct and is scored 1 point, with all other answers receiving a score of 0 points. The total score range is 0–28.

#### Teacher Characteristics and Teacher Training

The two teachers took part in the training and control activities, depending on their group assignment. We constructed and proposed training based on the TiM Project principles and methods, and supervised the teacher during the application of the TiM Project methods with two meetings during the school year. We also developed a control activity, similar to training for scheduling and methods, but without promoting the implementation of mentalization within the standard educational strategies. Meta-cognitive and meta-emotional skills of the two teachers were evaluated prior to the study by administering the MESI (Moè et al., 2010), a set of questionnaires that assess working practice, teaching satisfaction, positive and negative emotions related to work, positive and negative emotions related to the role of teacher, teaching strategies, self-efficacy and upgradeability (see **Table 1**). Both teachers showed values in line with the psychometric characteristics derived from the Italian validation of the measure. Specifically, all the scores were significantly distant from the critical thresholds identified for each scale and the two teachers’ values for each scale differ one from another appreciably less than one standard deviation.

**Table 1:** Value of the MESI scales for the two teachers.

Measures	WP	TSA	ERW+	ERRT+	ERW-	ERRT-	TS	*SE*	UP
TEACHER 1 (Training group)	4.08	5.80	4.20	3.92	1.53	2.06	3.6	8.00	8.5
TEACHER 2 (Control group)	4.12	6.00	4.23	4.15	1.65	1.76	4.13	8.29	8.88
CRITICAL THRESHOLDS (*SD*)	<3.68 (0.40)	<3.89 (1.10)	<2.87 (0.63)	<2.50 (0.71)	>2.38 (0.52)	>2.60 (0.59)	<2.99 (0.59)	<5.97 (1.06)	<5.92 (1.20)

*WP, working practice; TSA, teaching satisfaction; ERW+, positive emotions related to work; ERRT+, positive emotions related to the role of teacher; ERW-, negative emotions related to work; ERRT-, negative emotions related to the role of teacher; TS, teaching strategies; SE, self-efficacy; UP, upgradeability.*

#### Test Condition: the TiM Project Training

The aims of the TiM Project training were to introduce and to explain the key concepts and methods of the TiM Project, to involve the teacher in the direct experience of these methods, and to reflect together on the way to apply the TiM Project methods in the classroom with children. The TiM Project training was organized in two meetings, each lasting 3 h. At the end of the training, the teacher proposed the TiM Project methods to the classroom in the way the teacher liked, meeting the researcher for a supervision session on 2 days during the school year. Moreover, the teacher could ask for support at any time, contacting the researcher by e-mail or by the phone.

In the first meeting of the TiM Project training the researcher explained “The Thinking Brain and The Alarm Center,” which concern two concepts regarding brain functioning and are the basis of the TiM Project (Bak, 2012; Bak et al., 2015). Moreover, the researcher explained the importance of the ability to direct attention to one’s own thoughts in order to know one’s own mind. For each concept, activities and games were proposed to clarify explanations and to suggest possible activities to use with children. In the case of “The Thinking Brain and The Alarm Center,” the teacher had to draw a picture representing her brain on alert, then the teacher had to build a spotlight of attention with paper and use it to observe the world around. In both cases, reflection on the activities was promoted by the researcher.

In the second meeting, the term “resilience” was introduced and it was linked to the body-mind relationship. The activity proposed was the construction of a poster with a list of stressful situations of everyday life at school and the identification of the strategies that the teacher could use, with a focus on cognitive and emotional regulation strategies (involving the management of the alarm system). Moreover, the researcher introduced the TiM stories, such as the story of the “House of Thoughts” (Bak, 2012; Bak et al., 2015): a metaphor of the brain as a house of thoughts with the possibility to visit different rooms containing positive and negative thoughts (an example of the story is provided in the Appendix). To better understand the contents of this story, the teacher participated in a role play acting the role of a thought that inhabits one’s own brain.

At the end of this training the researcher guided a reflection on how to use the TiM Project methods with the children, and then the researcher delivered to the teacher the *TiM Project Manual* consisting of the Italian translation of the contents of the TiM Project website. During the following months, the researcher met the teacher twice to know how the teacher proceeded in applying the techniques, and to guide her in the preparation of new activities for the classroom. The teacher could also benefit from online or telephone support (advice, clarification, and suggestions) provided by the researcher over the entire length of the project.

#### Control Condition: the Non-mentalizing Training

The aims of the control condition training were to promote reflection about the teaching strategies that the teacher can apply in the classroom. More specifically, the focus was on the advantages and disadvantages of the traditional lecture method, and the strategies to support collaborative and cooperative learning. The control condition training was organized in two meetings.

In the first meeting the researcher explained the advantages and disadvantages of the traditional lecture method, and the teacher discussed professional experiences with this method and on the role as tutor of collective reasoning.

In the second meeting, the properties and the differences of cooperative learning (Johnson and Johnson, 2012) and collaborative learning (Nagata and Ronkowski, 1998) were discussed. The researcher explained the strategies and methods to encourage the active participation of students, and to promote the responsibility of each pupil in the working group. As in the TiM Project training, the teacher could also benefit from online or telephone support (advice, clarification, and suggestions) provided by the researcher over the entire length of the project.

### Procedures

The research was organized in three steps.

Step 1: Children were tested for their mentalization and theory of mind abilities (pre-test, 5 weeks after the beginning of the school year), and teachers participated in the TiM Project training or the control group training.

Step 2: Each teacher applied the training that she participated in, and teachers received supervision both in the presence of the researcher (two meetings during the school year, respectively, 2 and 4 months from the pre-test) and remotely (on-line).

Step 3: Children were re-tested for their mentalization and theory of mind abilities (post-test, 5 weeks at the end of the school year).

Each child was interviewed individually in two sessions of about 20–25 min each in a quiet room at the school. The procedure was identical for each participant. All tasks were administered in a fixed order. No feedback was given to children’s answers in the pre-test and in the post-test sessions. Teachers were trained in a room of the school. Informed parental consent was obtained for the children, and informed consent was obtained from each teacher. The three steps of the research were conducted by independent researchers. The research was conducted according to APA ethical standards and was approved by the local ethics committee.

## Results

**Table 2** reports the descriptive statistics for the explored variables at pre-test and post-test for the two groups; namely, the total scores of each task as they have been used in subsequent analyses unless otherwise specified.

**Table 2:** Descriptive statistics of the explored variables for children.

	TiM project condition							Control condition						
	Pre			Post				Pre			Post			
Measures	*n*	*M*	*SD*	*n*	M	*SD*	*p*	*n*	*M*	*SD*	*n*	*M*	*SD*	*p*
SS	23	8.70	2.49	23	9.91	2.31	0.002	23	7.87	1.77	23	9.65	2.67	0.001
PS	23	5.04	1.77	23	5.57	1.38	0.208	23	5.00	1.54	23	5.65	1.37	0.126
RMET-C	23	19.09	3.83	23	19.65	3.08	0.483	23	18.13	3.07	23	17.52	2.57	0.359
Third FBT	23	0.56	0.79	23	1.65	0.57	0.016	23	0.56	0.79	23	0.61	0.78	0.285
Second FBT SP	18	0.67	0.91	22	1.23	0.97	0.004	18	0.94	0.99	21	1.47	0.87	0.003
Second FBT LP	22	1.45	0.74	22	1.95	0.21	0.002	23	1.35	0.93	23	1.74	0.69	0.106
MT ON	23	4.00	1.86	23	3.39	1.73	0.225	23	3.61	1.92	23	3.04	1.55	0.178
MT R	23	6.70	2.10	23	9.91	2.02	0.000	23	7.09	1.86	23	7.83	1.99	0.077
MT OP	23	4.26	1.68	23	1.70	1.40	0.000	23	4.30	1.49	23	4.00	1.76	0.455

*n, number of participants; M, mean score; SD, standard deviation; SS, Strange Stories; PS, physical stories; RMET-C, Reading the mind in the eyes test-child version; third FBT, third-order false belief task; second FBT SP, second-order false belief task say prediction version; second FBT LP, second-order false belief task look prediction version; MT ON, mentalizing task overly negative style; MT R, mentalizing task rational style; MT OP, mentalizing task overly positive style. *P*-values refer to the *t*-test for paired samples between pre- and post-conditions within each group.*

We conducted some preliminary analyses to verify the homogeneity of the groups for the considered variables at the pre-test session. The *t*-test for independent samples did not show any statistically significant differences between the children assigned to the TiM Project training group and the children assigned to the control training group (all *p*s > 0.05).

Next, we performed a GLM for repeated measures for each variable explored (Mentalizing task, second and third order false belief tasks, Strange Stories, RMET) with time (pre-test and post-test) as the within-subjects factor and training groups (TiM Project and control) as the between-subjects factor. In order to test the training effect. The results showed a significant main effect of time for LP and SP tasks, Strange Stories, and the OP and R Mentalizing styles. Performance increased over the time for second order false belief LP: [*F*_(1,44)_ = 9.85, *p* = 0.003, $\eta_{p}^{2}$ = 0.186, 𝜃 = 0.866]; SP: [*F*_(1,44)_ = 9.40, *p* = 0.004, $\eta_{p}^{2}$ = 0.227, 𝜃 = 0.845] and Strange Stories understanding [*F*_(1,44)_ = 27.46, *p* = 0.001, $\eta_{p}^{2}$ = 0.384, 𝜃 = 0.999]. Furthermore, the OP style decreased [*F*_(1,44)_ = 30.1, *p* = 0.000, $\eta_{p}^{2}$ = 0.406, 𝜃 = 1], whereas the R styles increased [*F*_(1,44)_ = 37.30, *p* = 0.000, $\eta_{p}^{2}$ = 0.459, 𝜃 = 1]. The results also showed a significant interaction between time and training groups for the third FBT [*F*_(1,44)_ = 24.18, *p* = 0.001, $\eta_{p}^{2}$ = 0.392, 𝜃 = 0.999] and the Mentalizing task [*F*_(2,43)_ = 4.48, *p* = 0.017, $\eta_{p}^{2}$ = 0.173, 𝜃 = 0.737].

**FIGURE 1:**
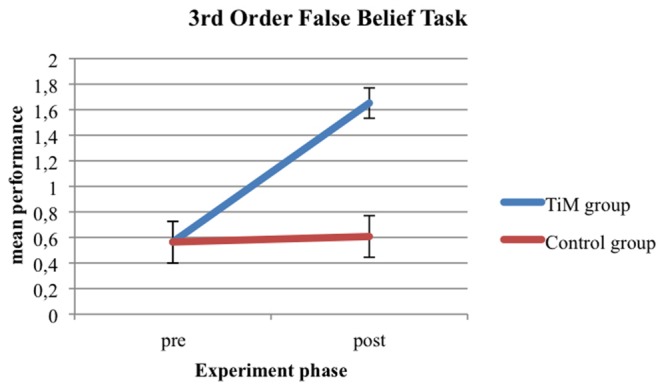
**Third-order FBT performance for TiM Project group and control group at pre-test and post-test**.

More specifically, pairwise comparisons revealed that, for the third FBT, the children in the TiM Project training group showed a significantly higher post-test performance compared to the post-test performance of children in the control training group [*F*_(1,44)_ = 26.62, *p* = 0.001, $\eta_{p}^{2}$ = 0.377, 𝜃 = 0.999] (see **Figure 1**).

**FIGURE 2:**
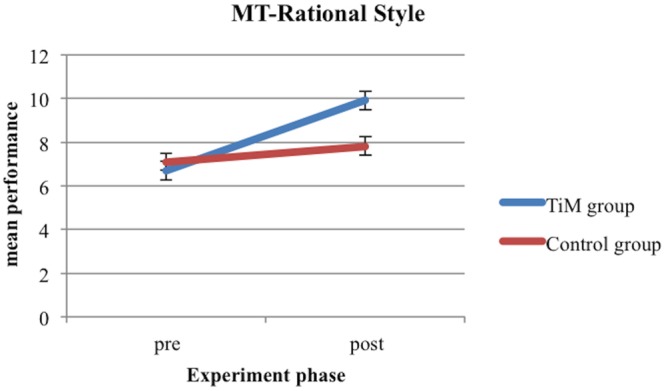
**MT-Rational style for TiM Project group and control group at pre-test and post-test**.

**FIGURE 3:**
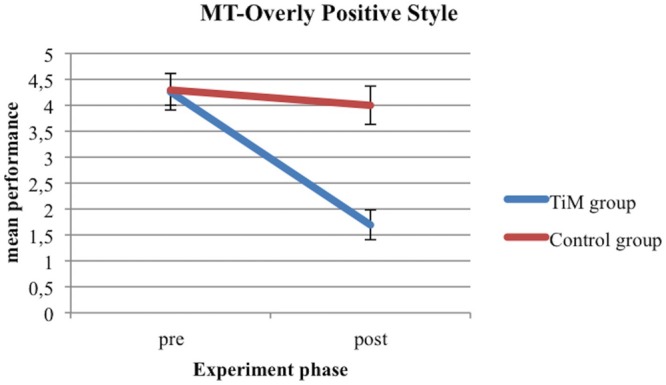
**MT-Overly positive style for TiM Project group and control group at pre-test and post-test**.

With regard to the Mentalizing task, in the post-test the children in the TiM Project training group showed a significantly higher performance on the R style of the task [*F*_(1,44)_ = 12.44, *p* = 0.001, $\eta_{p}^{2}$ = 0.220, 𝜃 = 0.932] and a significantly lower performance on the OP style of the task [*F*_(1,44)_ = 24.24, *p* = 0.001, $\eta_{p}^{2}$ = 0.355, 𝜃 = 0.998] than children in the control training group (see **Figures 2** and **3**).

## Discussion

The present research preliminarily explored the efficacy of the TiM Project training on mentalization performance in 10-year-old pupils. To this aim, we tested children’s cognitive, affective, and social components of theory of mind as well as mentalizing styles. The training succeeded in promoting specific elements of mentalistic ability. We will discuss these results starting from disentangling this specificity from the mere time effect that occurred with regard to some variables.

Performance on the second-order false belief tasks and Strange Stories showed an increase over time. The understanding of the second level of recursivity begins to be successfully overcome around 7 years of age, although in his review Miller (2009) pointed out that the available studies indicate that this type of task continues to improve until pre-adolescence. This period appears to be a sensible one also for the development of the comprehension of ambiguous social situations—here measured through the Strange Stories—where mentalization is implied. On the contrary, this was not the case for the capacity to “read” the mind through the eyes, because performance on average was already well developed and the RMET did not improve with time. The rational attributional style and the overly positive style also changed with time: the former increasing and the latter decreasing in respective scores. However, while the improvement in second order false belief understanding and ambiguous social situations understanding seems not to depend on the TiM project training, third order false belief understanding and the changes in mentalizing styles appear to be significantly supported by the training itself.

As for the comprehension of the third level of recursivity, the presence of the training effect could be interpreted in terms of efficacy of the teacher’s intervention in the pupils’ zone of proximal development (ZoPed), although no classroom observations were taken. In fact, this action pulls the comprehension from very low levels to intermediate ones. The same does not happen in the case of the second order false belief tasks (LP and SP). Considering together the results about the false belief understanding, the ZoPed acts on the comprehension of the third level of recursivity similar to what time does with the second level of recursivity. The absence of the effect of time and training on the RMET is not surprising; in fact, the average performance is already medium-high in the pre-test session. Furthermore, it is in line with the performance of slightly older subjects (see for example Sharp, 2008, in which a sample of children with an average age equal to 11 obtained a mean performance of 17.96 on the RMET). So, we can hypothesize that the time frame considered was not sufficiently long enough in order to have an effect on this ability. Kaland et al. (2008) showed that the performance of a sample with an average age of 15.6 obtained a mean performance score of 23.16. In addition, the training did not have more of an effect by being more focused on metacognitive abilities than affective aspects directly implied in the RMET.

With regard to the training effect on the OP and R mentalizing styles, the literature shows that the critical age for a change in attributional style is 7–11 year-olds. Indeed, from 4 to 7 years of age children generally attribute an overly positive judgment to peers about their behavior, whereas from 8 years on the attributional style becomes more rational and more congruent with objective indicators (Damon and Hart, 1991; Berndt and Burgy, 1996; Harter, 1999). Furthermore, Sharp et al. (2007) also corroborated the presence of a critical period for variations in the attributional style of children ages 7 to 11 years old, suggesting that these changes are closely related to the ability to take the perspective of others in complex social situations. The participants in the present study are in the top margin of this critical range. Therefore, it is plausible that they have already undergone the developmental changes. This fact would explain the absence of the effect of time. On the contrary, the training may promote a change in the ZoPed, anticipating a change that it is likely to appear later. This explanation is also consistent with the work of Meins et al. (2002, 2006) showing that the maternal proclivity to consider the child as an individual with mental states and not just as the bearer of needs supports the acquisition of the child’s mentalistic skills according to longitudinal dynamics. This attitude, otherwise known as mind-mindedness, offers the child the opportunity to engage actively with his or her own and others’ mental states, and to understand the mentalistic attitudes that people have toward the world. This relational competence is exercised in the ZoPed and, through the process of internalization, affects the child’s ability to interact mentally with partners (Laranjo et al., 2014; Meins et al., 2014).

The fact that the teacher had attended the TiM Project training and had used the training in the classroom increased the children’s capacity to apply a rational attributional style to other’s mind, and decreased the tendency to use an overly positive attributional style. This result supports the efficacy of the TiM Project training application in the classroom, and suggests that teachers involved in it can help their pupils to increase an attributional style that can act as a potential protective factor against psychopathology (Baumeister et al., 1996). In fact, although it has been observed that children have the tendency to misperceive the thoughts, feelings, and intentions of others (O’Connor and Hirsch, 1999), it seems that emotional disorders are associated with specific attributional styles in childhood (Ingram et al., 1998). Sharp et al. (2007) affirmed that an overly positive attributional style (i.e., estimating the judgment of peers on themselves in an overly positive way) combined with a lack of a rational attributional style (i.e., an objective evaluation of other people’s thoughts) is associated with symptoms of externalizing disorder (as individuated by teachers). Additionally, Hughes et al. (1997, 2001), David and Kistner (2000), and Brendgen et al. (2004) linked together the aggression in primary school children, the over-estimation of peer acceptance, and the tendency to idealize the perception of one’s own qualities.

Although Mentalizing tasks and the Strange Stories have the common aim of investigating theory of mind understanding in complex social situations, the performance on the Strange Stories was not affected by the training. This discrepancy can be explained by considering two aspects related to the tasks. The first one concerns the characteristics of the tasks in terms of instructions and test questions. In fact, theory of mind understanding is evaluated in the mentalizing task by asking the child to put him/herself in another person’s shoes, whereas the Strange Stories asks the child to explain another person’s behavior. The Mentalizing task requires a first person simulation of another’s mind, which is isomorphic to the way the content of the TiM project training is implemented by the teachers in the classroom. The second aspect that can explain the above-mentioned discrepancy regards the structure of the test questions. In the Mentalizing task, children are faced with a forced choice among three possible answers (corresponding to the three mentalizing styles), while in the Strange Stories children are faced with open questioning. Due to its intrinsic metacognitive features, the TiM project training appears to be more suited for promoting a form of mentalization more coherent with the forced choices format than with the open questioning format. Finally, the social situations proposed in the Strange Stories imply the understanding of numerous components of theory of mind that the TiM Project training does not involve [for example, the case of irony and sarcasm (Massaro et al., 2014)].

This study, despite offering some interesting evidence supporting the implementation of mentalizing strategies, presents some methodological issues that need to be carefully evaluated for the interpretation of the results. First, the sample size is limited: only two classes were compared. Accordingly, only two teachers, for the training and the control groups, were involved. Secondly, classroom observations should be implemented in order to evaluate the teacher’s strategies applied to support mentalization and to identify situations in which the TiM Project can be used with the greatest impact. Finally, although teachers did not differ in metacognitive and metaemotional skills, their mentalizing abilities were not directly evaluated in the pre-training phase. The possibility that the significant variation observed in children’s mentalizing abilities depended on past differences between the teachers cannot be excluded. However, it is important to note that teachers were involved in training and the children tested for mentalizing abilities during the 5th year of primary school (i.e., after 4 years of interaction with their teachers), and that in the pre-training phase there were no differences in children’s mentalizing abilities between the two groups of children.

Future research should replicate these results with better management of these issues. Furthermore, given that recently Bak et al. (2015) evaluated the welfare of the operators involved in the training, the inclusion of teacher evaluations may prove to be a significant element. Finally, the inclusion of a wider sample of teachers that allows the exploration of possible covariates of the implementation of mentalizing strategies is highly desirable.

## Conclusion

This study provides some preliminary evidence to support the validity of the TiM Project. It is likely that a teacher who has an increased understanding of mental functioning, and who can talk about it in the classroom, is able to help children to increase their mentalistic skills. In particular, children improve their mentalizing attributional style (from overly positive to rational), which consequently can reduce the risk of psychopathology, increase the level of recursive thinking in cognitive theory of mind, and increase learning to reason at a third level of recursivity. Although these findings require further investigation, they remain promising about the idea that the creation of a mentalizing community promotes the mentalization abilities of its members, evaluating for the first time this efficacy on children’s competencies. These communities can be consistently regarded as the extension of ZoPed within which, as just mentioned, the mother uses the mind-mindedness (Laranjo et al., 2014; Meins et al., 2014) to support child’s mentalization. Similarly, the teachers, specifically trained, will accompany children in the acquisition of more and more effective and socially adaptive mentalist abilities (Meins et al., 2013).

## Author Contributions

AV, DM, IC, FSI, EL, EB, and AM conceived and designed the experiments. AV, DM, IC, FSI, EL, and EB performed the experiments. AV, DM, IC, FSI, EL, and AM analyzed the data. AV, DM, IC, FSI, EL, EB, and AM wrote the paper.

## Conflict of Interest Statement

The authors declare that the research was conducted in the absence of any commercial or financial relationships that could be construed as a potential conflict of interest.

## Footnotes

1 http://myresilience.org

## APPENDIX

### Example of TiM Project Story

#### The Story of The House of Thoughts^2^

In some way, we may say that our thoughts live inside our heads. Imagine that your thoughts live in a house with many rooms where you can wander around and discover them. When you discover thoughts you are using the world’s finest tool – your attention, which is a kind of spotlight. When you throw light on a thought, you spot it and discover it. Thereafter you can shift your attention and discover another thought. The House of Thoughts has plenty of rooms – a number of exciting thoughts may live in one room, perhaps some sad or angry thoughts live in another room and various happy thoughts live in a third room. From The House of Thoughts, your thoughts can call you if they want to be discovered. This may be really exciting and good, but could be irritating too – especially if the thoughts are annoying and they keep knocking all the time, trying to take charge over your attention. In the case, where you have sad or angry thoughts that take charge and force you into their room all the time, you might end up believing there are no exciting or happy thoughts to be found anywhere and that is not much fun…. Yet this is not the case at all. All the happy and exciting thoughts are just waiting in other rooms in the House of Thoughts, waiting for you to discover them with your attention. Maybe there even are tools to be found in one room that could be used to fix some other thoughts in another room in the house. There may also be thoughts in a room who need to be left in peace, so they won’t disturb you to much. If you often go to explore The House of Thoughts with your attention, then it becomes easier to be in charge with your thoughts.
